# hUMSC transplantation restores ovarian function in POI rats by inhibiting autophagy of theca-interstitial cells via the AMPK/mTOR signaling pathway

**DOI:** 10.1186/s13287-020-01784-7

**Published:** 2020-07-03

**Authors:** Xueyan Lu, Hongchu Bao, Linlu Cui, Wenqian Zhu, Lianshuang Zhang, Zheng Xu, Xuejing Man, Yongli Chu, Qiang Fu, Hongqin Zhang

**Affiliations:** 1 College of Basic Medicine & Institute of Reproductive Diseases, Binzhou Medical University, Yantai, 264003 Shandong China; 2 College of Basic Medicine, Binzhou Medical University, Yantai, 264003 Shandong China; 3 Department of Clinical Medicine, Yantai Yuhuangding Hospital, Yantai, 264000 Shandong China

**Keywords:** Primary ovarian insufficiency, Theca-interstitial cells, Human umbilical cord-derived mesenchymal stem cells, Autophagy, Oxidative stress, AMPK

## Abstract

**Background:**

Previous studies of primary ovarian insufficiency (POI) have focused on granulosa cells (GCs) and ignored the role of theca-interstitial cells (TICs). This study aims to explore the mechanism of the protective effects of human umbilical cord-derived mesenchymal stem cells (hUMSCs) on ovarian function in POI rats by regulating autophagy of TICs.

**Methods:**

The POI model was established in rats treated with cisplatin (CDDP). The hUMSCs were transplanted into POI rats by tail vein. Enzyme-linked immunosorbent assay (ELISA) analysis, hematoxylin and eosin (HE) staining, and immunohistochemistry were used to measure the protective effects of hUMSCs. The molecular mechanisms of injury and repairment of TICs were assessed by immunofluorescence, transmission electron microscope (TEM), flow cytometry (FCM), western blot, and quantitative real-time polymerase chain reaction (qRT-PCR).

**Results:**

In vivo, hUMSC transplantation restored the ovarian function and alleviated the apoptosis of TICs in POI rats. In vitro, hUMSCs reduced the autophagy levels of TICs by reducing oxidative stress and regulating AMPK/mTOR signaling pathway, thereby alleviating the apoptosis of TICs.

**Conclusion:**

This study indicates that hUMSCs protected ovarian function in POI by regulating autophagy signaling pathway AMPK/mTOR.

## Background

Primary ovarian insufficiency (POI) is an encompassing disorder associated with conditions ranging from irregular ovulation to premature menopause and covers all non-physiological declines in ovarian reserves [1, 2]. POI refers to the loss of ovarian activity in women before the age of 40 and is manifested as amenorrhea or oligomenorrhea, accompanied by elevated gonadotropin and decreased estradiol (E_2_) levels [3]. It has reported that chromosomal abnormalities, immune factors, metabolic abnormalities, surgery, radiotherapy, drugs, and especially chemotherapy drugs can induce POI [4–9]. Cisplatin (CDDP), one of the commonly used chemotherapeutic drugs, has been reported to trigger POI and result in decreased hormonal levels of an acceleration in follicle loss and an enhancement of ovarian aging [10, 11]. Recently, mesenchymal stem cells (MSCs) have been considered an ideal cell line for POI treatment due to its low immunogenicity, strong proliferative capacity, and an isolation method which is less ethically controversial [12–15]. We have previously demonstrated the recovery of ovarian function after MSCs transplantation [16], but the ovarian protection mechanisms involved with MSCs transplant have not been fully investigated. At present, the literatures available on this topic have focused on the role of granulosa cells (GCs) on oocytes without reports on the effects of theca-interstitial cells (TICs) on GCs and oocytes, despite a potentially important role of TICs in this process. TICs show an increased sensitivity to gonadotropins and enhanced the effect of growth factors in the blood of follicle development and promotion of the synthesis of E_2_ in GCs via testosterone (T) secretion to play an overall important role in the development and atresia of follicles [17, 18]. Therefore, given the lack of data and potential significance of TICs, one of the goals of this study was to focus on the growth and development of TICs affected by human umbilical cord-derived mesenchymal stem cells (hUMSCs).

Reactive oxygen species (ROS) produced during metabolic activity caused by oxidative stress is one of the most important influencing factors which induces ovarian aging [19, 20]. At the same time, amp-activated protein kinase (AMPK), a sensor of cellular energy levels, is activated by the increment of the AMP/ATP ratio, which is induced during the oxidative stress. The activation of AMPK can inhibit mammalian target of rapamycin (mTOR) signaling [21] to activate autophagy which can result from high ROS levels produced by oxidative stress to control cellular energy homeostasis [22, 23]. However, it remains unclear that whether autophagy is involved in the recovery of ovarian function following hUMSC transplantation in POI. Therefore, in this study, we investigated the effects of hUMSCs on the autophage of TICs in POI rats and found that hUMSCs can reduce the oxidative stress, thereby reducing the autophagy level through AMPK/mTOR pathway.

## Methods

### Experimental animals

Eighty female Wistar rats (6 weeks old) were purchased from the Jinan Pengyue experimental animal breeding Co, Ltd. (Jinan, China). All experimental procedures were approved by the Binzhou Medical University Institutional Animal Care and Use Committee and this study was conducted in accordance with the National Laboratory Animal Care and Use research committee guidelines.

### Chemicals

CDDP (Meilunbio, China) was dissolved in warmed distilled water (DW) at a stock concentration of 3.33 mM and added to TICs cultures at a final concentration of 0–60 μM. The ROS inhibitor, N-acetyl-l-cysteine (NAC, Sigma, USA), was dissolved in DW at a stock concentration of 80 mM and added to TICs cultures at a final concentration of 5 mM. The AMPK inhibitor, Compound C (Selleck, USA), was dissolved in alcohol at a stock concentration of 5 mM and added to TICs cultures at a final concentration of 5 μM. 3-Methyladenine (3MA, Selleck, USA) was dissolved in DW at a stock concentration of 10 mM and added to TICs cultures at a final concentration of 5 μM.

### Establishment of POI model

The POI model was generated by daily intraperitoneal injections of CDDP (2 mg/kg, dissolved in saline) for 7 days. The rats were randomly divided into four groups (*n* = 20/group): (1) control, intraperitoneal injection with the saline only; (2) POI, intraperitoneal injection with CDDP; (3) POI + hUMSCs, POI rat injected in tail vein with phosphate buffer saline (PBS) containing 2 × 10^6^ hUMSCs 7 days after CDDP injection; and (4) POI + PBS group, POI rat injected in tail vein with PBS only. Seven days following hUMSC treatment, the rats were euthanized for further studies.

### Serum hormone measurement

Serum samples from hearts were collected and stored at − 80 °C prior to assay. Serum levels of E_2_, luteinizing hormone (LH), and follicle-stimulating hormone (FSH) were determined with use of an ELISA kit (Mlbio, China).

### Ovarian follicle counting and morphological analyses

Ovarian tissues were collected, fixed in 4% paraformaldehyde, and embedded in paraffin for hematoxylin and eosin (HE) stain. The slides were imaged and analyzed using light microscopy. Follicles were classified as primordial, primary, secondary, or atresia follicles in accordance with a method described previously [24].

### Isolation and characterization of hUMSCs

The hUMSCs isolation procedure was approved by the Research Ethics Committee of the Yantai Yuhuangding Hospital. The phenotypes of hUMSCs were confirmed with cell surface markers expression. Cells were incubated with fluorescein isothiocyanate-conjugated mouse anti-human CD34, CD45, CD73, CD90, CD44, and HLA-DRmAb (BD Biosciences and Invitrogen, USA) and detected using of flow cytometry (BD Biosciences, USA). Additional verification of the hUMSCs phenotype was established by detection of their ability to differentiate into adipocytes and osteoblasts [25].

### Isolation, culture, and identification of ovarian TICs

Ovarian tissues were collected from the rats (3 weeks old) as described previously [26]. Tissues were collected in Leibovitz’s L-15 medium (Gibco, USA) containing 10% fetal bovine serum (FBS, AusGeneX, Australia), 1% 100 U/mL streptomycin sulfate and 100 U/mL penicillin G. The adipose and connective tissues were removed. The GCs were isolated by puncturing the follicles using a sterile syringe needle under a stereoscope (Olympus, Japan). Remaining tissues were washed three times with PBS, digested in collagenase II type (Biosharp, China) and McCoy’s 5A medium (Gibco, USA) at a ratio of 1:1 for 1 h at room temperature, then centrifuged at 1000 rpm at 37 °C for 5 min and washed twice with McCoy’s 5A medium [27]. TICs were then resuspended in McCoy’s 5A medium containing 10% FBS, 1% 100 U/mL streptomycin sulfate, and 100 U/mL penicillin G and maintained in an incubator at 37 °C in a humid environment with 5% CO_2_ for 3 days. The first passage of TICs was used in all experiments.

The phenotype of TICs was confirmed by detecting the expression of specific cell surface marker Cyp17a1 and T [28]. The expression of follicle-stimulating hormone receptor (FSHR) and Cyp17a1 on the TICs were assessed by immunofluorescence. TICs were incubated overnight at 4 °C with rabbit anti-rat FSHR antibody (1:150; abcam, UK) and Cyp17a1 (1:150, abcam, UK). After washing with PBS, TICs were incubated for 1 h at 37 °C with a secondary biotinylated mouse anti-rabbit IgG antibody (1:500, abbkine, USA). TICs were then washed in PBS and incubated for 20 min at 37 °C with DAPI dye liquor (Solarbio, China). The staining of FSHR and Cyp17a1 were observed with use of fluorescent microscopy (Leica, Germany). ELISA was used to detect E_2_ and T levels in the TICs supernatant according to the manufacturer’s instruction (Mlbio, China).

### CCK-8 cell viability assay

The effect of different concentrations of CDDP on TICs viability was determined using of CCK-8 kits (Meilunbio, China). Cells (5000 TICs/well) were seeded in a 96-well plate (Corning, USA) for overnight incubation. Following adherence of the cells, the culture medium was replaced with medium containing CDDP (0–60 μm). CCK-8 cells (10 μl) were added to the cell medium for 1 h at different time points. The absorbance was determined using of an ELISA reader at 450 nm. The value was calculated and analyzed according to manufacturer’s instructions.

### mRFP-GFP-LC3 fluorescent staining

The mRFP-GFP-LC3 fluorescent staining procedure was used to detect autophagy levels. TICs were transfected with mRFP-GFP-LC3 adenovirus (Hanbio, China) for 48 h, then washed three times with PBS and incubated with different concentrations of CDDP (20 μm) for different times. After three washes with PBS, cells were fixed in 4% paraformaldehyde at 37 °C for 20 min. To visualize autophagy, cells were imaged using confocal microscopy (Carl Zeiss AG, Germany) under × 400 magnification. The number of red and green dots on each cell were counted.

### Inhibitor experiment

After achieving 80% confluence, TICs were seeded in 6-well plates (1 × 10^5^cells/well). Cells were divided into six groups according the different treatments for 20 h: (1) control group, untreated medium; (2) CDDP group, CDDP (20 μM) alone; (3) CDDP + NAC group, CDDP + NAC (5 mM); (4) CDDP + 3MA group, CDDP + 3MA (5 μM); (5) CDDP + compound C group, CDDP + compound C (5 μM); and (6) CDDP + hUMSCs group, CDDP + hUMSC supernatants.

### Transmission electron microscopy (TEM)

TEM was used to assess the autophagy structure within TICs in vitro. TICs were grown in 6-well plates up to 90% confluence. After washing, cells were fixed in 2.5% glutaraldehyde followed by 1% osmium tetroxide at 4 °C for 90 min. Cells were then dehydrated in alcohol and embedded in epoxy resin. Cell slides were stained with uranylacetate and lead citrate and examined under TEM (JEOL, Japan).

### Cellular ROS production in TICs

To investigate ROS production in TICs, the cells were incubated in 100 μl PBS containing 150 ng of 2,7-dichlorofluorescein-diacetate (DCFH-DA, Njjcbio, China) at 37 °C for 30 min. After washing, the cells were resuspended in PBS and then detected with use of flow cytometry (FCM). The production of ROS in TICs was measured by fluorescence intensity following dichlorofluorescein (DCF) staining using flow cytometry (BD Biosciences, USA).

### Quantitative real-time polymerase chain reaction (qRT-PCR)

Total RNA was isolated from TICs using Trizol reagent (Ambion, USA) and reversed transcribed into cDNA using Transcriptor HiFi cDNA Synth (Roche, Germany). The primers for quantitative real-time polymerase chain reaction were as follows: Ampk forward primer: CAGCACCGGAGGTCATCTCA and reverse primer: GCACGTGCTCATCGTCGAA; Mtor forward primer: GCTTATCAAGCAAGCGACATCTCA and reverse primer: TCCACTGGAAGCACAGACCAAG; glyceraldehyde-3-phosphate dehydrogenase (GAPDH) forward primer: GGCACAGTCAAGGCTGAGAATG and reverse primer: ATGGTGGTGAAGACGCCAGTA. Quantitative real-time PCR was published by Light Cycler Fast Start DNA Master SYBR Green I Kit (Roche, Germany). The housekeeping gene, Gapdh, was used as an internal control.

### Western blot analysis

After harvesting of cells, TICs were lysed using radioimmunoprecipitation assay (RIPA) buffer. Protein concentrations were determined using of the bicinchoninic acid (BCA) assay (Solarbio, China). Proteins were separated using sodium dodecyl sulfate polyacrylamide gel electrophoresis (SDS-PAGE) and transferred to polyvinylidene fluoride (PVDF) membranes. After blocking with 5–7% skim milk, membranes were incubated overnight at 4 °C with anti-AMPK, anti-p-AMPK, anti-m-TOR, anti-p-mTOR (1:1000, CST, USA), anti-LC3B (1:2000, Abcam, UK), and anti-GAPDH (1:20,000, Proteintech, China) polyclonal antibodies. The membranes were then washed three times with TBS added with Tween 20 (TBST) and incubated with secondary antibodies (1:20,000) at 37 °C for 1 h. Expressions of proteins were measured with use of the enhanced chemiluminescence reagent (ECL) kit (Novland, China). Band densities were measured with use of ImageJ software.

### Immunohistochemistry

Apoptosis within ovarian tissue was detected by anti-cleavage caspase-3 antibody staining on the paraffin slide. Ovarian sections fixed on the paraffin were incubated with rabbit primary polyclonal antibodies against rat cleaved caspase-3 (1:150, Abcam, UK) at 4 °C overnight. The slides were then incubated with the biotinylated secondary antibody (1:500) at 37 °C for 30 min and developed with diaminobenzidine (DAB) as chromogen and then counterstained with hematoxylin. The German immunoreactive score (IRS) was used to analyze staining results as described previously [29].

### Autophagy detection with MDC staining

Dansylcadaverine (MDC) is a fluorescent dye that detects specific markers of the autophagosome. TICs were washed and stained with 10 μl MDC (Solarbio, China) for 25 min in the dark. Cell staining was then assessed with use of laser scanning confocal microscopy (Leica, Germany).

### Immunofluorescence

Immunofluorescence was used for determination of LC3 expression. TICs were washed with PBS, fixed in 4% paraformaldehyde for 30 min, and incubated with anti-LC3B (1:500, Abcam, UK) at 4 °C overnight, followed by incubation with Goat anti-Rabbit IgG, Alexa Fluor 488 (Invitrogen, USA) at 37 °C for 1 h. DAPI was then used to stain nuclei, which were then imaged using fluorescent microscopy (Echo, USA).

### Statistical analysis

Data were expressed as the means ± standard deviation (SD) and analyzed using the SPSS 21.0 statistic software program. One-way analysis of variance (ANOVA) was used to assess overall differences among the groups with post hoc comparisons. *P* value of < 0.05 was considered statistically significant.

## Results

### hUMSCs phenotype characterization

The hUMSCs isolated from fresh umbilical cords formed clone spheres after 7–10 days. The cells displayed a fibroblast-like morphology (Additional file: Supplemental figure 1b) and were induced into osteocytes stained with Alizarin Red S staining (Additional file: Supplemental figure 1c) and adipocytes stained with Oil red O staining (Additional file: Supplemental figure 1d). Results of flow cytometry analysis confirmed the presence of positive expressions of mesenchymal progenitor markers (CD73, CD44 and CD90) and negative expressions of hematopoietic cell surface markers (CD34, CD45, and HLA-DR) (Additional file: Supplemental figure 1a). The demonstration of these characteristics confirmed that hUMSCs had been successfully isolated as reported previously [4].

### Ovarian function recovery following hUMSC transplantation in POI rats

**Fig. 1:**
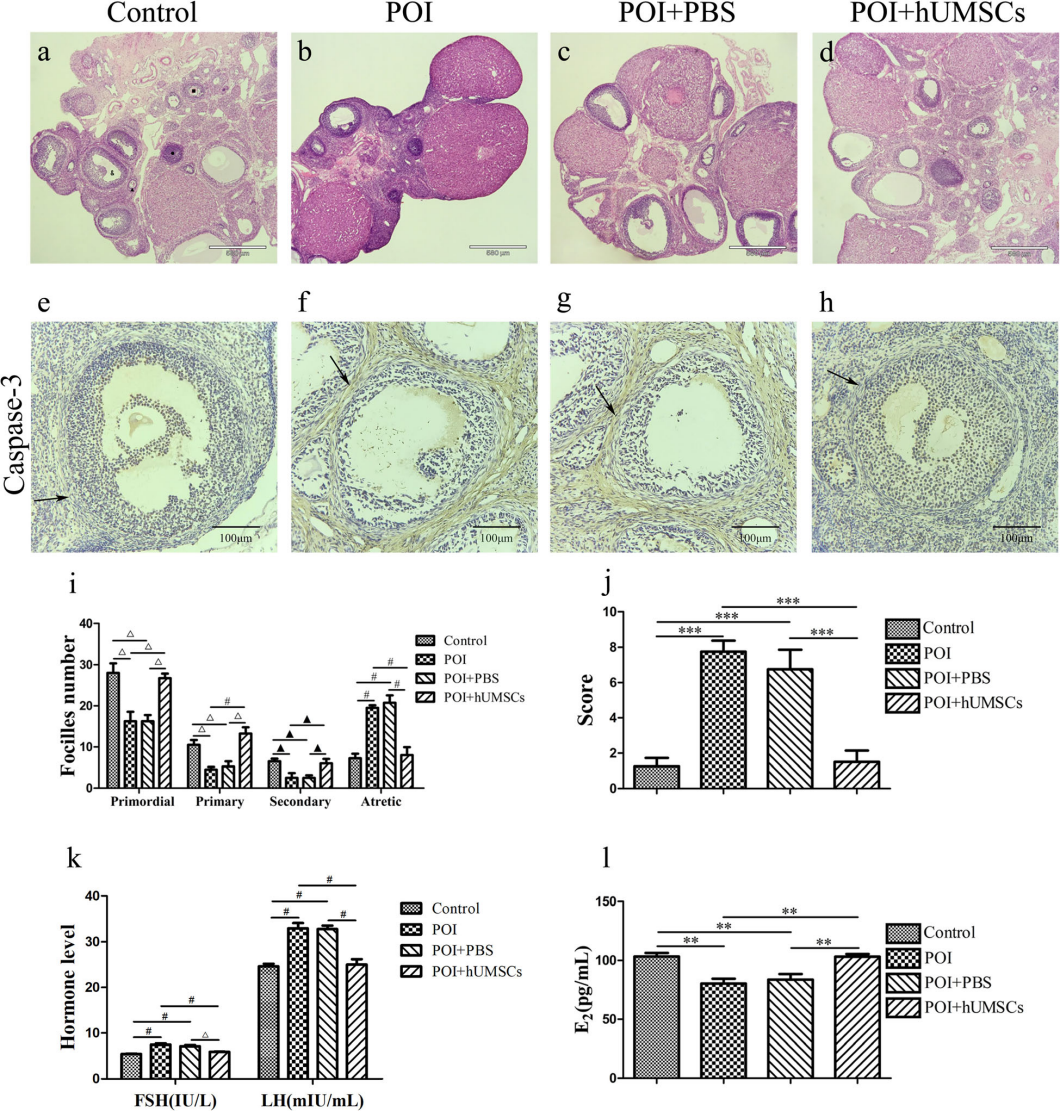
Effects of hUMSC transplantation on ovarian tissue histopathology, apoptosis, follicle counts and blood levels of hormone. **a**–**d**, × 40, Ovarian tissue histopathology was determined with use of HE staining (triangle indicates the primordial follicle, square indicates the primary follicle and the secondary follicle, circle indicates the atretic follicle). **e**–**h**, × 200, Caspase-3 staining was examined by immunohistochemistry shown as brown with the cell nucleus being stained blue. Arrow indicates the theca-interstitial cell layer. **i** Summary of follicle counts from ovaries within each group. **j** Intensity of caspase-3 staining quantification within each group. **k**, **l** Summary of serum E_2_, FSH, and LH release within each group. Data are expressed as the means ± SD, *P < 0.05, **P < 0.01, ***P < 0.001, black triangle indicates *P* < 0.05, white triangle indicates *P* < 0.01, and ^#^*P* < 0.001. hUMSCs, human umbilical cord-derived mesenchymal stem cells; HE, hematoxylin and eosin; E_2_, estradiol; FSH, follicle-stimulating hormone; LH, luteinizing hormone

To assess the effects of hUMSC transplantation on ovarian function in CDDP-induced POI rats, the ovarian morphology, follicle count, and serum levels of FSH, LH, and E_2_ were determined. We found that ovaries in the POI and POI + PBS groups showed more atrophic than that observed in the control and POI + hUMSCs groups. Also, ovaries of POI rats showed a significant reduction in follicle counts at different stages of development, especially primordial follicles (Fig. 1a–d). After hUMSC transplantation, the number of normal follicles was significantly increased and the number of atresia follicles greatly reduced, compared with the POI and POI + PBS groups (Fig. 1i). With regard to hormonal levels, the POI and POI + PBS groups showed lower levels of E_2_ and higher levels of FSH and LH, compared with the control and POI + hUMSCs groups (Fig. 1k, l). These data demonstrated that a successful generation of a POI animal model was established and hUMSCs restored the morphology of the ovary of the POI rats.

We further examined the effects of hUMSC transplantation on apoptosis of ovarian cells using of immunohistochemistry staining of caspase-3. The data showed that caspase-3 positive cells were distributed within the theca-interstitial layer of the ovaries within POI and POI + PBS rats (Fig. 1e–h, j). Following hUMSC transplantation, the number of apoptotic cells was significantly decreased. These findings demonstrate that recovery of ovarian function may be mediated by decreasing the apoptosis in POI rats following hUMSC transplantation.

### TICs characterization

**Fig. 2:**
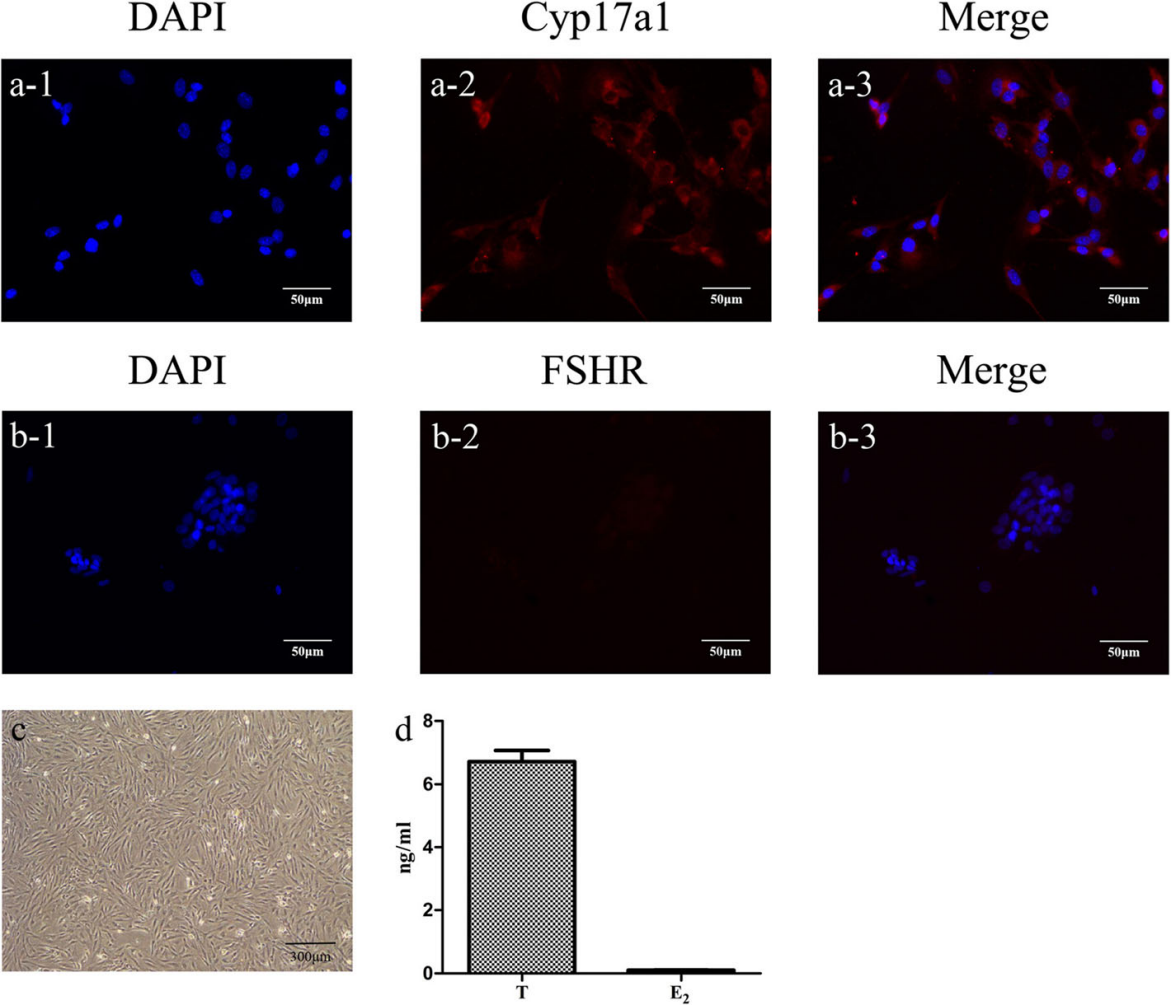
Immunostaining with Cyp17a1 and FSHR to characterize TICs (× 200). **a1**–**a3** Cyp17a1 expression in TICs-blue fluorescence with DAPI nuclear staining and red fluorescence with Dylight 549 staining. **b1**–**b3** FSHR expression in TICs-blue fluorescence with DAPI staining and red fluorescence with Dylight 549 staining (scale bar = 100 μm). **c** Microscopic morphology of TICs. **d** Levels of T and E_2_ in culture medium of TICs. Data are expressed as the means ± SD. FSHR, follicle-stimulating hormone receptor; TICs, theca-interstitial cells; DAPI 4, 6-diamino-2-phenyl indole; T, testosterone; E_2_, estradiol

The morphology of isolated TICs was shown in Fig. 2c. The cells showed fibroblasts appearance after adherence. Cyp17a1 is specifically expressed in TICs [30, 31], while the FSHR is only expressed in GCs [32]. After immunostaining with FSHR or Cyp17a1, TICs were stained red by Dylight 549 and nuclei stained blue with DAPI. Characterization of TICs was confirmed by the observation of positive staining with Cyp17a1 and negative staining of FSHR as shown in Fig. 2a and b. These results demonstrate that the isolated cells were TICs, but not GCs. Moreover, TICs can secrete large amounts of T but not E_2_ [33]. The higher levels of T and lower expressions of E_2_ within the supernatant further substantiate the identification of the TICs (Fig. 2d).

### Effects of CDDP treatment on autophagy in TICs

**Fig. 3:**
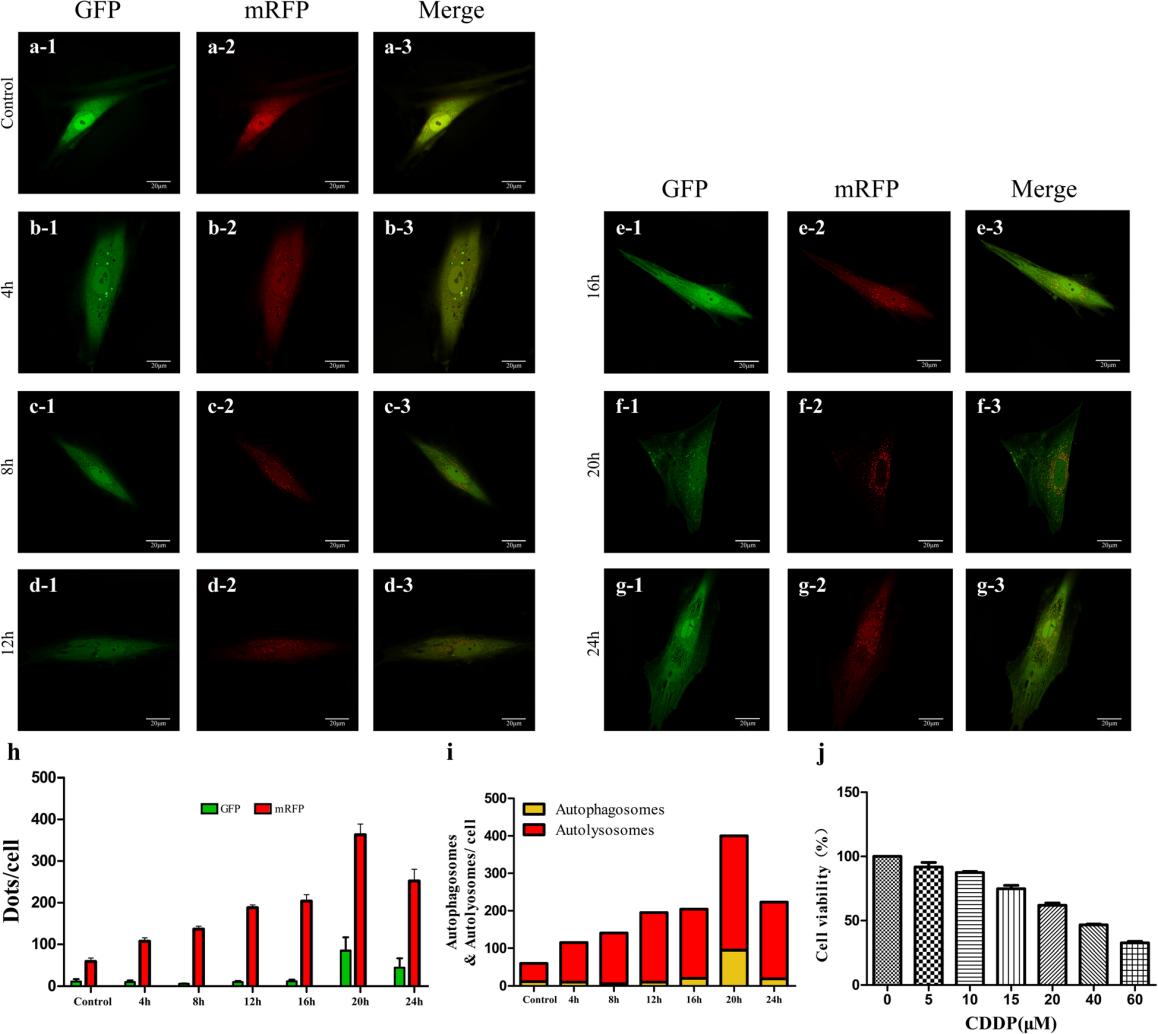
Effect of CDDP on autophagy and cell viability of TICs. **a**–**g** Representative fluorescence images of mRFP-GFP-LC3 at different exposure times from 4 to 24 h—green fluorescent with GFP staining and red fluorescence with mRFP staining. **h** Summary of dot distribution per cell from GFP and mRFP staining. **i** Average number of autolysosomes as indicated by red dots only and autophagosomes with both red and green dots within cells. **j** Cell viability of TICs as a function of treatment with varying CDDP concentrations. Data are expressed as the means ± SD. CDDP, cisplatin; TICs, theca-interstitial cells

We chose CCK-8 and mRFP-GFP-LC3 assays to determine the autophagy levels. Concentrations of CDDP assessed using CCK-8 and exposures time were selected based on previous reports [34–36]. TICs were incubated with varying concentrations of CDDP (0, 5, 10, 15, 20, 40, or 60 μM) for 24 h (Fig. 3j) to detect cell viability using CCK-8 analysis. According to this result, we chose 20 μM for the following experiment. Then, TICs were incubated with 20 μM CDDP at different time points (0 h, 4 h, 8 h, 12 h, 16 h, 20 h, 24 h) to evaluate autophagy flux using mRFP-GFP-LC3. Autophagy flux is a dynamic process that assesses the degree of autophagy and is considered one more comprehensive means to evaluate the degree of autophagy than that achieved by directly detecting autophagosomes [37] as mRFP-GFP-LC3 staining can distinguish autophagosomes from autolysosomes [38].

The GFP fluorescence is quenched by a decrease in pH after entering the lysosome. Under such conditions, pH stability of mRFP is greater than that of GFP, because mRFP still fluoresces after entering the autolysosome. Autophagosomes can be distinguished from autophagolysosomes by observing the number of green and yellow dots within the merged images. Red dots overlying green dots are autophagosomes and appear yellow in the merged image, while red dots that are not covered by green dots in the merged image are autophagolysosomes. The time points used in the treatment of TICs and performance of mRFP-GFP-LC3 staining are shown in Fig. 3a–i. CDDP treatment produced a dose-dependent effect on cell viability as shown in Fig. 3j.

### Effect of CDDP on ROS production and AMPK/mTOR pathway activation in TICs co-cultured with hUMSC supernatant

**Fig. 4:**
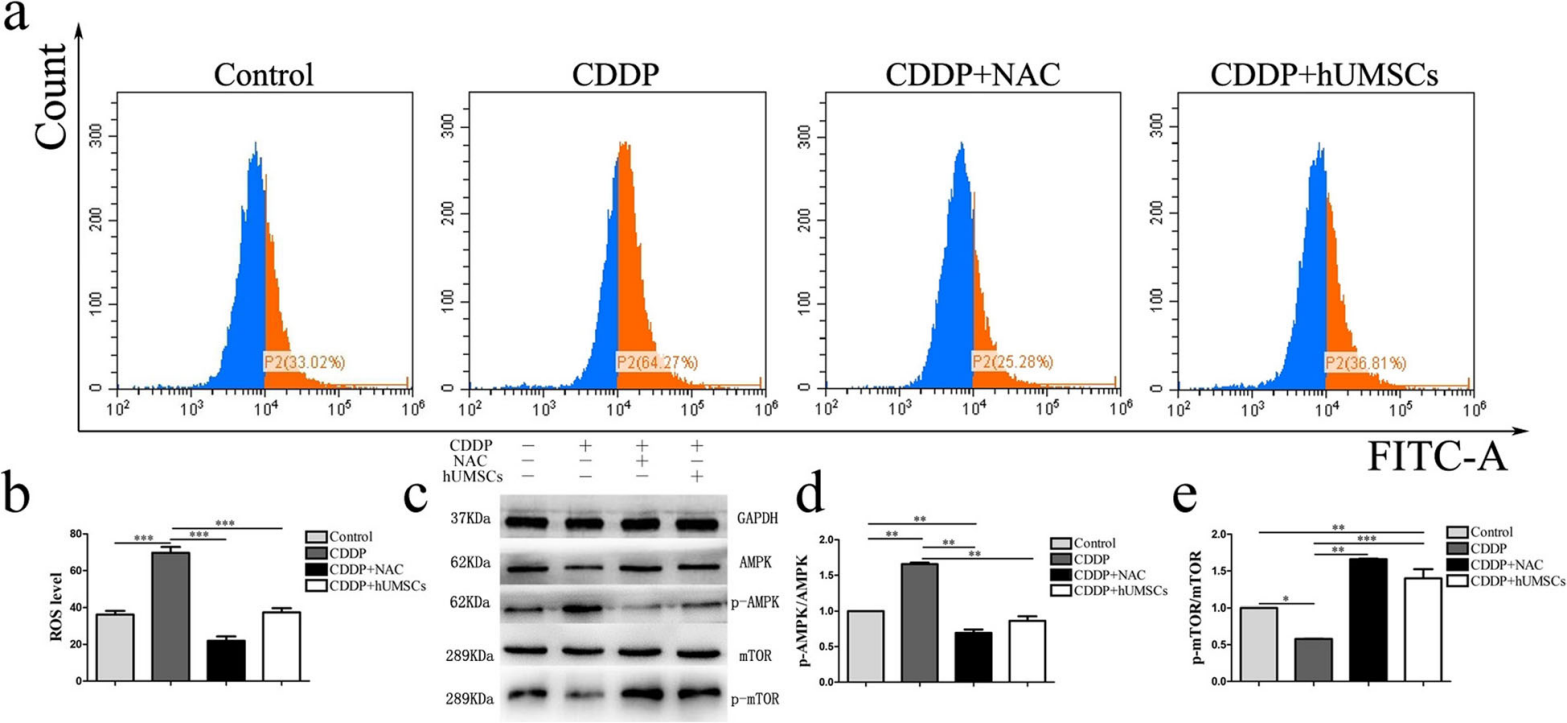
Effects of co-culture of hUMSC supernatant with TICs on CDDP-induced ROS production and AMPK/mTOR pathway activation. **a** Representative flow cytometric plot of ROS production within the different groups—red area represents positive ROS production. **b** Quantification of intracellular ROS production within each group. **c**–**e** Western blot analysis indicating signal protein activation of AMPK, p-AMPK, mTOR, and p-mTOR in TICs following antioxidant NAC treatment and hUMSC supernatant co-culture. Data are expressed as the means ± SD, *P < 0.05, **P < 0.01, ***P < 0.001. hUMSCs, human umbilical cord-derived mesenchymal stem cells; TICs, theca-interstitial cells; CDDP, cisplatin; ROS, reactive oxygen species; AMPK, Amp-activated protein kinase; mTOR, mammalian target of rapamycin; NAC, N-acetyl-l-cysteine

Cellular ROS production in TICs resulting from CDDP was determined with DCFH-DA staining using FCM after co-culturing with hUMSC supernatants. NAC, an inhibitor of ROS, was added as a positive control. As shown in Fig. 4a, ROS production in the CDDP group was significantly increased compared with the control group. In contrast, intracellular levels of ROS were significantly decreased in CDDP groups that were co-cultured with hUMSCs or NAC treatment (Fig. 4b). Based on the western blot analysis (Fig. 4c–e), protein levels of p-AMPK/AMPK in the CDDP group were significantly increased, compared with the control group. Moreover, co-culturing with either NAC or hUMSC supernatant significantly inhibited the activation of AMPK signaling pathway. However, p-mTOR/mTOR showed opposite trend compared with p-AMPK/AMPK. These results suggest that hUMSC treatment significantly reduced oxidative stress in CDDP-treated TICs via AMPK/mTOR signaling pathway.

### Inhibition of hUMSC treatment on CDDP-induced autophagy

**Fig. 5:**
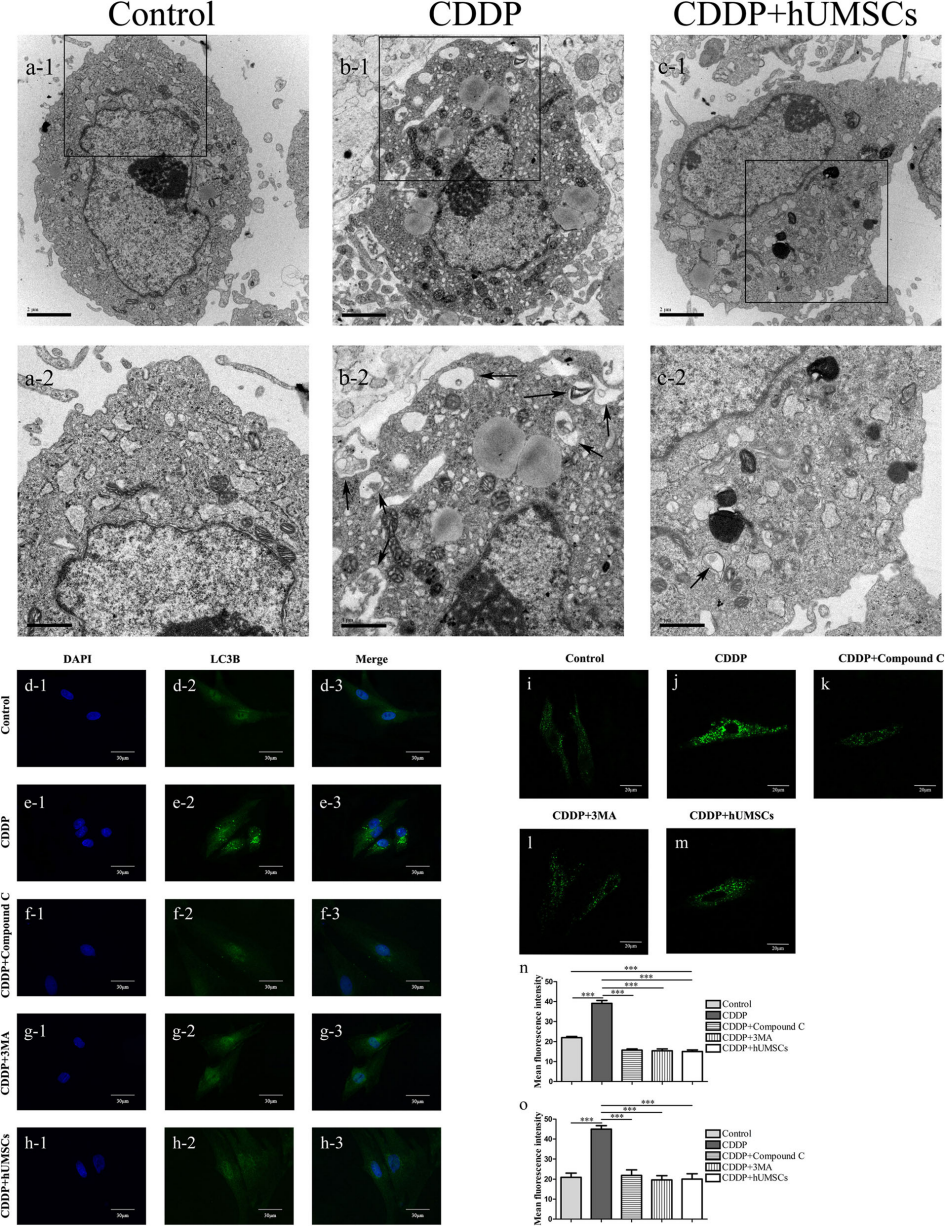
hUMSCs co-culture with TICs inhibit autophagy in TICs induced by CDDP. **a**–**c** TEM Images show normal ultrastructure with few autophagy structures in the Control and CDDP + hUMSCs groups. Within the CDDP group, most autophagy structures are diffusely located within the cytoplasm—black arrow indicates autophagy structure. **d**–**h** Representative immunofluorescence images of TICs as shown with specific autophagy marker staining of LC3B (green fluorescent) and nuclear DAPI staining (blue fluorescence) within each group. **n** Quantification of average fluorescent intensities with LC3B staining within each group. **i**–**m** TICs were stained with MDC to determine autophagy levels within each group. **o** Quantification of average fluorescent intensities with MDC within each group. Data are expressed as the means ± SD, ***P < 0.001. hUMSCs, human umbilical cord-derived mesenchymal stem cells; TICs, theca-interstitial cells; CDDP, cisplatin; DAPI, 4,6-diamino-2-phenyl indole; MDC, dansylcadaverine

**Fig. 6:**
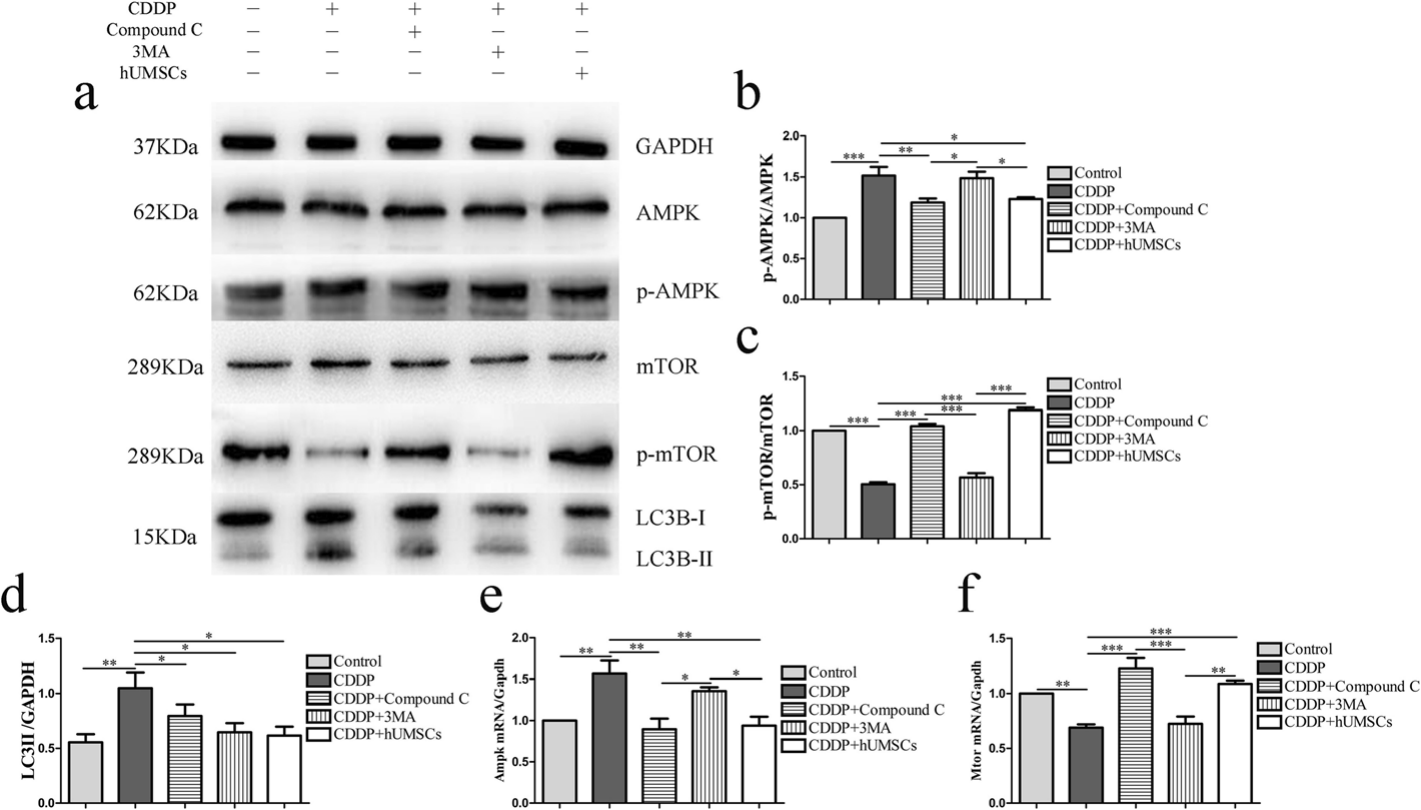
hUMSCs co-culture with TICs inhibit CDDP-induced autophagy in TICs through the AMPK/mTOR signaling pathway. **a** Protein expression of AMPK, mTOR and LC3B following 24 h of hUMSCs co-culture with TICs with or without Compound C or 3MA as determined using western blot analysis. GAPDH was used as an internal control to quantify protein amounts. **b**–**d** Quantification of phosphorylated AMPK, mTOR and LC3B expression levels within each group. **e**, **f** Analysis of ratios of RNA levels for Ampk/Gapdh and Mtor/Gapdh within each group. Data are expressed as the means ± SD, *P < 0.05, **P < 0.01, ***P < 0.001. hUMSCs, human umbilical cord-derived mesenchymal stem cells; TICs, theca-interstitial cells; CDDP, cisplatin; AMPK, Amp-activated protein kinase; mTOR, mammalian target of rapamycin; 3MA, 3-methyladenine

To investigate whether hUMSCs would alter CDDP-induced autophagy, we examined the number of autophagy structures, including autophagosomes and autophagolysosomes in vitro by using TEM. As shown in Fig. 5a–c, there was an increasement of autophagy structures within the CDDP-induced POI group. Following hUMSC treatment, the presences of autophagy structures were significantly reduced. As the level of LC3B-II is related to amount of autophagosomes, LC3B-II serves as a reliable marker of autophagy [39]. Therefore, TICs were stained with MDC and LC3 to determine the extent of autophagy in TICs with different treatment conditions (Fig. 5d–m). Based on fluorescence intensity, the data showed that autophagy levels in CDDP-treated cells significantly increased, compared with that of all other groups. While, the expression of LC3B-II was significantly inhibited following treatment with the autophagy inhibitor (3MA), AMPK inhibitor (Compound C) and hUMSC supernatant (Fig. 5n). Similar results were observed with MDC staining (Fig. 5o). In addition, AMPK, mTOR, and autophagy-related protein (LC3) expressions were analyzed by western blot and qRT-PCR in order to confirm whether AMPK/ mTOR pathway was involved in CDDP-induced autophagy (Fig. 6a–f).

### TICs apoptosis

**Fig. 7:**
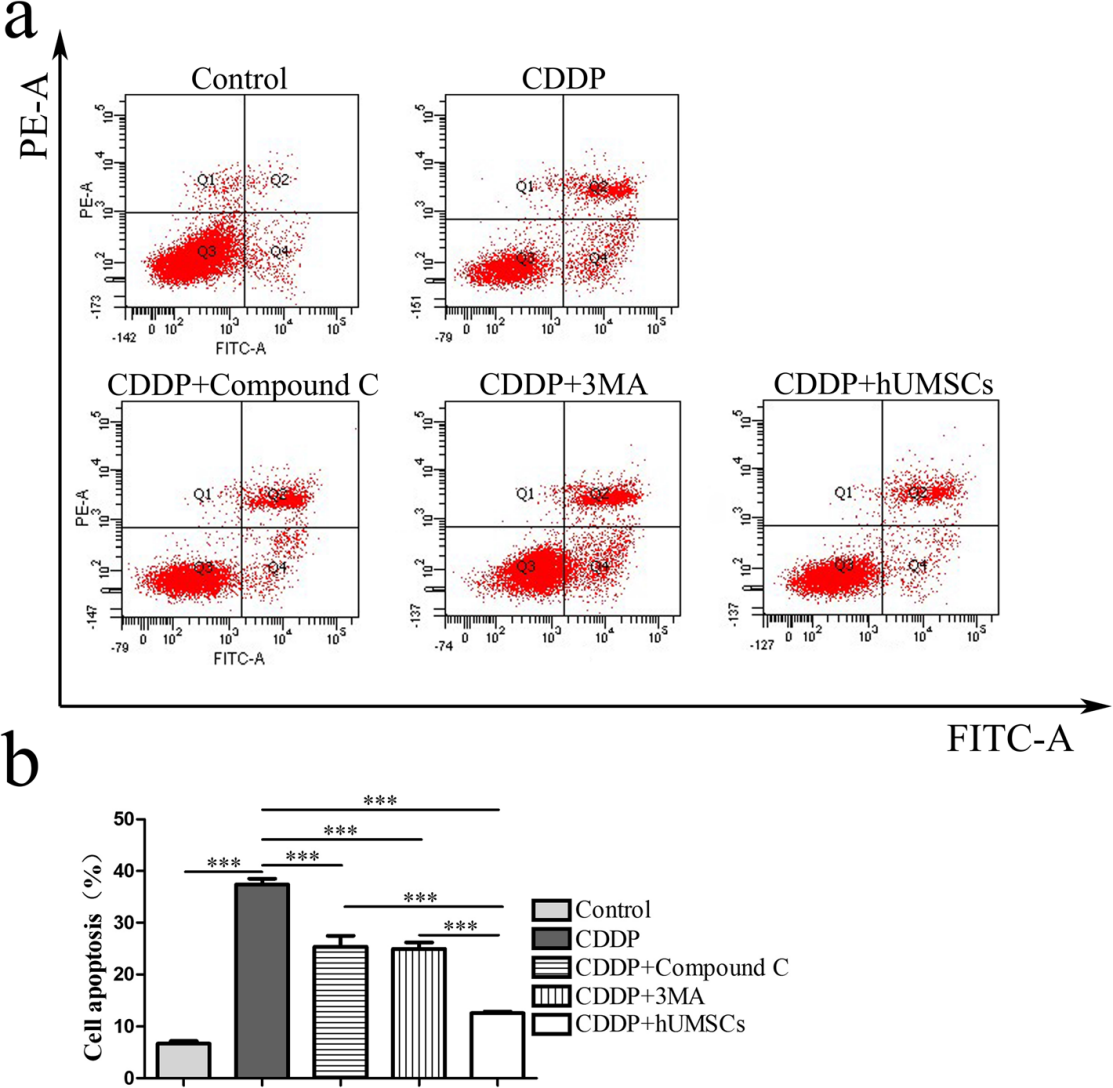
Effects of autophagy inhibitors on TICs apoptosis were determined using flow cytometry analysis. **a** Representative flow cytometry plots were obtained following treatment with 3MA, Compound C, and hUMSCs. **b** Quantification of apoptotic TICs within each group. Data are expressed as the means ± SD, ***P < 0.001. TICs, theca-interstitial cells; 3MA, 3-methyladenine; hUMSCs, human umbilical cord-derived mesenchymal stem cells

To further establish whether autophagy was involved in CDDP-induced TICs apoptosis, the apoptosis of TICs was examined using of flow cytometry (Fig. 7a). As shown in Fig. 7b, CDDP treatment produced a significant increase in apoptosis compared with the control group. Following treatment with 3MA or Compound C, the rate of apoptosis was significantly decreased, compared with that of the CDDP treatment alone group. The number of apoptotic cells within the hUMSCs group was significantly lower than that of the CDDP group treated with Compound C or 3MA. These data suggest that the apoptosis resulting from CDDP may be regulated by autophagy. Accordingly, inhibition of autophagy can reduce TICs apoptosis, and hUMSCs may serve as an effective means to produce such effects.

## Discussion

In this study, we investigated the effects of hUMSCs in a CDDP-induced rat model of POI and its underlying mechanisms. We found that hUMSC transplantation reduced cell apoptosis and improved ovarian function in CDDP-induced POI in rats; In vitro, the apoptosis and autophagy of TICs induced by CDDP could be reduced with Compound C, 3-MA, and hUMSCs, but maximal protective effects were observed in hUMSC treatment group. These results suggest that hUMSCs could alleviate POI injury by inhibiting the TICs apoptosis through reducing autophagy.

Decreased ovarian function in POI is related to the loss of resting follicles and decreased biological ability of atresia follicles [40]. TICs play an important role in folliculogenesis and provide nutrients and hormones to GCs through the basement membrane via vessels. Moreover, some growth factors are secreted from TICs, which can protect GCs from apoptosis [32, 41, 42]. Taken together, these findings suggest an important role for TICs in follicular atresia. Interestingly, the POI resulting from CDDP [4, 10, 11] involves induction of apoptosis, which leads to an impairment in overall female reproductive capacity [43]. However, apoptosis is not the sole factor for follicular atresia. As D’Herde K and others observed autophagic cell death in atresia follicles in 1996, but the underlying mechanisms of this process have not been investigated. Autophagy is closely related to cell death and plays an important role in embryonic development and ovarian diseases. CDDP induces a large number of cell apoptosis and tissue damage by stimulating oxidative stress [7, 44, 45], which activates autophagy through AMPK/mTOR signaling pathway [36]. Therefore, we determined whether the apoptosis of TICs in CDDP-induced POI rats involves changes of autophagy levels stimulated by oxidative stress.

It has been reported that MSCs isolated from amniotic fluid, amniotic membranes and placenta exert protective effects in POI induced by chemotherapy [46, 47]. In contrast, hUMSCs are ideal cell source in various treatments due to low immunogenicity, a strong proliferative capacity, absence of immune rejection [13, 14, 48]. It has been reported that MSCs transplantation can repair myocardial infarction by reducing autophagy [49], but the role of hUMSCs in TICs within POI remains unclear. Therefore, we investigated the potential role of hUMSCs to restore ovarian function in POI rats by regulating autophagy of TICs to reduce apoptosis.

In the CDDP-induced POI rats, our data showed that hUMSC transplantation enables recovery of ovarian function and increases follicle numbers. Within immature follicles, apoptosis mainly occurs in the theca-interstitial layer. Interestingly, after hUMSC transplantation, the number of apoptosis cells was significantly decreased within the theca-interstitial layer, suggesting that TICs play an important role in the occurrence of POI. A related component in this process is that of ROS, which induces oxidative stress and autophagy through AMPK/mTOR. In specific, AMPK can indirectly promote autophagy by inhibiting mTOR activity [50, 51]. In this study, we found that TICs co-cultured with hUMSC supernatant reduced ROS level which is resulted from CDDP-induced oxidative stress, thereby reducing the activity of AMPK, and consequently reduces the autophagy. In this way, our findings suggest that hUMSCs reduced CDDP-induced autophagy by inhibiting ROS production, reducing AMPK activity, and activating mTOR activity.

To address the issue of the relationship between autophagy and apoptosis in TICs after hUMSC treatment, we examined the apoptosis of TICs following treatment with AMPK or an autophagy inhibitor. The results showed that both inhibitors were effective in reducing the CDDP-induced apoptosis rate, but the maximal effectiveness was obtained in response to co-cultured with hUMSC supernatant. As inhibition of autophagy can reduce apoptosis in CDDP-induced damage, it indicates that hUMSCs may reduce the rate of apoptosis by inhibiting autophagy through AMPK/mTOR. The identification of this cascade of events provides a useful working model to explain the recovery of ovarian function in POI rats following hUMSC transplantation.

This study indicates that hUMSCs protect ovarian function in POI by regulating autophagy of TICs. These data provide novel information to elucidate the mechanisms of ovarian function recovery following hUMSC transplantation, which serves as a foundation for the development of more effective strategy in the treatment of POI patients.

## Conclusion

The data in our study shows that hUMSC transplantation can result in the recovery of ovarian function in POI rats. This recovery is associated with autophagy of TICs in part through regulating ROS levels and inhibiting the AMPK/mTOR signaling pathway.

## Supplementary information

**Additional file 1: Supplemental figure 1** hUMSCs characteristics were confirmed by cell surface marker staining and cell differentiation ability. (a) Blue histograms represent negative control staining and red histograms expression of specific cell surface markers. (b) hUMSCs display a fibroblast-like morphology under light microscopy (40×). (c) Osteoblasts stained with Alizarin Red S that were positive showed a brown color, indicating calcium deposition (100×). (d) Accumulation of neutral lipid vacuoles by oil red O staining indicates adipogenesis as indicated by red (200×). hUMSCs human umbilical cord-derived mesenchymal stem cells.

## Data Availability

All data generated and/or analyzed during this study are included in this published article.

## Abbreviations

POI: Primary ovarian insufficiency
GCs: Granulosa cells
TICs: Theca-interstitial cells
hUMSCs: Human umbilical cord-derived mesenchymal stem cells
CDDP: Cisplatin
ELISA: Enzyme-linked immunosorbent assay analysis
HE: Hematoxylin and eosin
TEM: Transmission electron microscope
FCM: Flow cytometry
qRT-PCR: Quantitative real-time polymerase chain reaction
E_2_: Estradiol
MSCs: Mesenchymal stem cells
T: Testosterone
ROS: Reactive oxygen species
AMPK: Amp-activated protein kinase
mTOR: Mammalian target of rapamycin
DW: Distilled water
NAC: N-Acetyl-l-cysteine
3MA: 3-Methyladenine
PBS: Phosphate buffer saline
LH: Luteinizing hormone
FSH: Follicle-stimulating hormone
FSHR: Follicle-stimulating hormone receptor
FBS: Fetal bovine serum
DCFH-DA: 2,7-Dichlorofluorescein-diacetate
DCF: Dichlorofluorescein
GAPDH: Glyceraldehyde-3-phosphate dehydrogenase
RIPA: Radioimmunoprecipitation assay
BCA: Bicinchoninic acid
SDS-PAGE: Sodium dodecyl sulfate polyacrylamide gel electrophoresis
PVDF: Polyvinylidene fluoride
TBST: TBS added with Tween 20
ECL: Enhanced chemiluminescence reagent
DAB: Diaminobenzidine
IRS: Immunoreactive score
MDC: Dansylcadaverine
DAPI: 4,6-Diamino-2-phenyl indole
SD: Standard deviation
ANOVA: One-way analysis of variance
