# Supplementary figures and images for: hUMSC transplantation restores ovarian function in POI rats by inhibiting autophagy of theca-interstitial cells via the AMPK/mTOR signaling pathway
Source: Stem Cell Res Ther. 2020 Jul 3;11:268. doi: 10.1186/s13287-020-01784-7 (PMC7333437; doi:10.1186/s13287-020-01784-7)

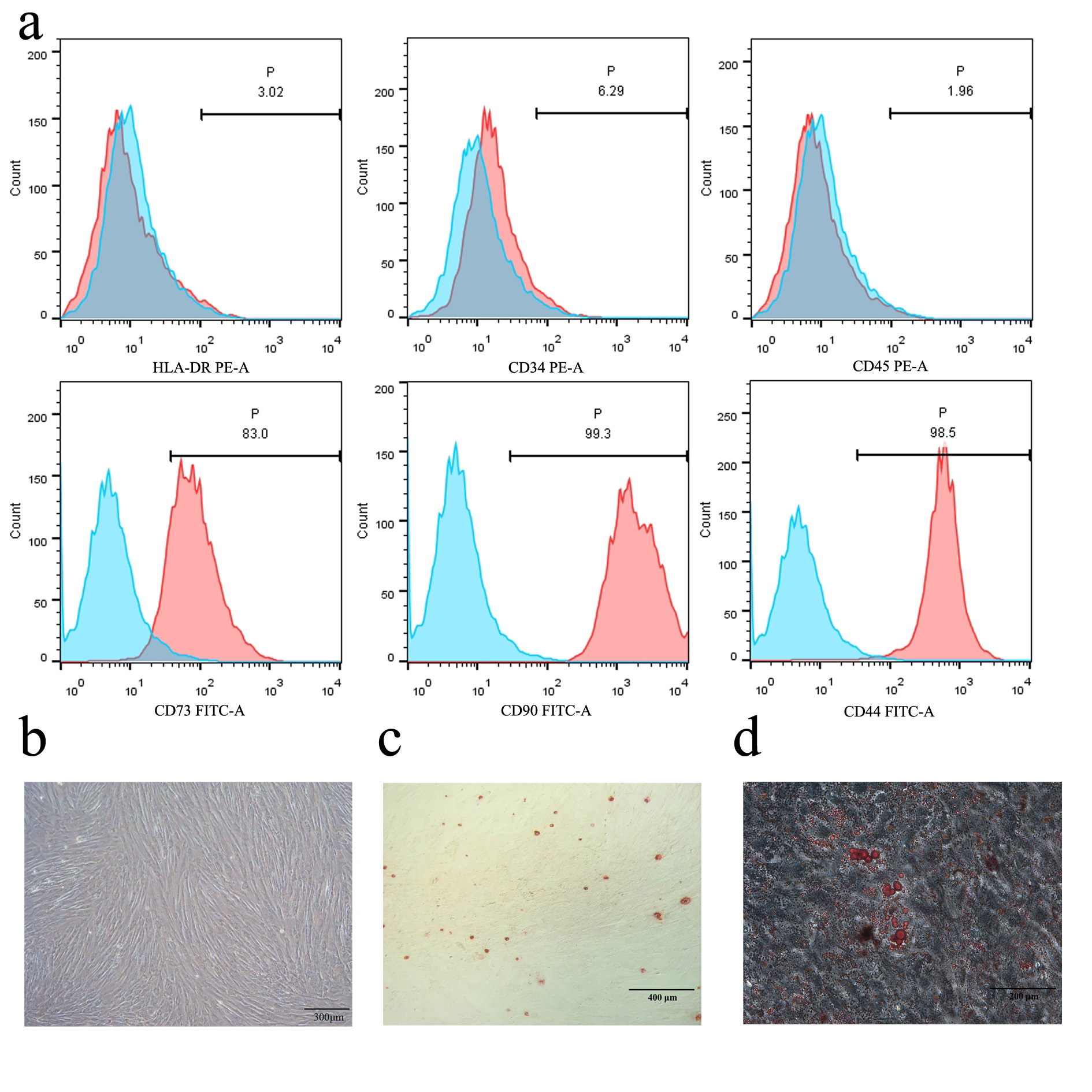

Supplement: Supplementary file 1 — Additional file 1: Supplemental figure 1 hUMSCs characteristics were confirmed by cell surface marker staining and cell differentiation ability. (a) Blue histograms represent negative control staining and red histograms expression of specific cell surface markers. (b) hUMSCs display a fibroblast-like morphology under light microscopy (40×). (c) Osteoblasts stained with Alizarin Red S that were positive showed a brown color, indicating calcium deposition (100×). (d) Accumulation of neutral lipid vacuoles by oil red O staining indicates adipogenesis as indicated by red (200×). hUMSCs human umbilical cord-derived mesenchymal stem cells. [file 13287_2020_1784_MOESM1_ESM.jpg]
